# AS-YOLO: Enhanced YOLO Using Ghost Bottleneck and Global Attention Mechanism for Apple Stem Segmentation

**DOI:** 10.3390/s25051422

**Published:** 2025-02-26

**Authors:** Na Rae Baek, Yeongwook Lee, Dong-hee Noh, Hea-Min Lee, Se Woon Cho

**Affiliations:** IT Application Research Center, Jeonbuk Regional Branch, Korea Electronics Technologies Institute (KETI), Jeon-Ju 54853, Republic of Korea; nrbaek@keti.re.kr (N.R.B.); thisis206@keti.re.kr (Y.L.); dhee.noh@keti.re.kr (D.-h.N.); lee10849@keti.re.kr (H.-M.L.)

**Keywords:** stem segmentation, ghost bottleneck, global attention mechanism, RGB-D sensor, agricultural automation robot, intelligent agriculture

## Abstract

Stem removal from harvested fruits remains one of the most labor-intensive tasks in fruit harvesting, directly affecting the fruit quality and marketability. Accurate and rapid fruit and stem segmentation techniques are essential for automating this process. This study proposes an enhanced You Only Look Once (YOLO) model, AppleStem (AS)-YOLO, which uses a ghost bottleneck and global attention mechanism to segment apple stems. The proposed model reduces the number of parameters and enhances the computational efficiency using the ghost bottleneck while improving feature extraction capabilities using the global attention mechanism. The model was evaluated using both a custom-built and an open dataset, which will be later released to ensure reproducibility. Experimental results demonstrated that the AS-YOLO model achieved high accuracy, with a mean average precision (mAP)@50 of 0.956 and mAP@50–95 of 0.782 across all classes, along with a real-time inference speed of 129.8 frames per second (FPS). Compared with state-of-the-art segmentation models, AS-YOLO exhibited superior performance. The proposed AS-YOLO model demonstrates the potential for real-time application in automated fruit-harvesting systems, contributing to the advancement of agricultural automation.

## 1. Introduction

**Figure 1:**
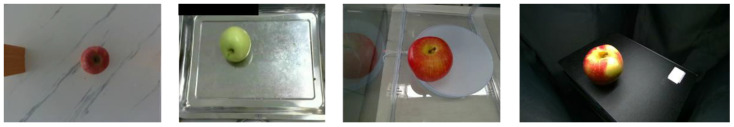
Sample images from AppleStem-Segmentation (AS-Seg) dataset used in the experiments.

Recently, advancements in agricultural technology have become crucial for improving efficiency and reducing costs through mechanized harvesting techniques. Apples, one of the world’s most economically significant crops, present a challenge for automated systems in accurately identifying both the fruit and its stem, which is essential for maintaining freshness and minimizing damage during postharvest processing [1]. Despite technological progress, stem removal remains labor-intensive, requiring significant labor within a short period. Automation of this process requires accurate segmentation techniques that utilize computer vision and deep-learning technologies [2,3]. Rapid advancements in computer vision technology have been crucial in addressing the challenge of accurately segmenting fruit and stems. Traditional detection methods [4,5,6,7,8,9,10] primarily focus on identifying the location or boundaries of fruits, however, accurately recognizing smaller regions such as stems remains a significant challenge. As depicted in Figure 1, stems occupy a considerably smaller area than fruits, requiring higher precision for recognition. To overcome these limitations, deep learning-based detection and segmentation models have evolved progressively, offering more refined and precise solutions to segmentation tasks.

Existing deep learning-based studies on apple and stem segmentation, such as those employing multimodal approaches [11] or two-step convolutional neural networks (CNN) [12,13], have shown promising results in terms of accuracy. However, there are significant limitations in terms of processing speed. In particular, two-stage segmentation architecture models such as Mask R-CNN [14] are not suitable for real-time application due to the substantial computational burden at each stage. In [13], Mask R-CNN was applied to experiments across various environments and achieved high segmentation accuracy. However, its processing time was still 1.18 s per image, which is not enough for real-time applications. Moreover, multimodal models that integrate RGB and near-infrared (NIR) imagery [11] showed high accuracy but it took time to align images between the two modalities. Any misalignment between the modalities can lead to degraded performance. Furthermore, RGB and NIR cameras are required to acquire images, and in the case of the NIR camera additional lighting device is required, making them less practical for deployment in real-world agricultural environments. In this way, as the module becomes more complex, the performance improves, but it does not meet the critical requirement of real-time processing, which is essential for agricultural automation.

In this paper, we propose AS-YOLO, an enhanced YOLO model developed to optimize the balance between performance and processing time for solving the problem of stem segmentation in apples. We reduce the number of parameters and improves computational efficiency through ghost bottleneck module [15]. Additionally, the Global Attention Mechanism (GAM) [16] is utilized to enhance the feature extraction capability of the model, enabling finer differentiation between stems and fruits. To validate the performance of the proposed AS-YOLO model, we used the AppleStem-Segmentation (AS-Seg) dataset [17], which includes both a self-constructed dataset and open datasets, as shown in Figure 1. This dataset was collected under various environmental conditions and includes diverse apple data. These variations enhance the reliability of the experimental results, ensuring the robustness of the model across different scenarios. Through comparative experiments using the dataset, we confirmed that AS-YOLO showed superior performance than existing methods.

Therefore, the proposed AS-YOLO model demonstrates the potential for real-time application in stem removal tasks performed by fruit-harvesting robots, laying the groundwork for significant advancements in agricultural automation. This approach is expected to substantially improve the efficiency of labor-intensive fruit harvesting work, further contributing to the automation of the agricultural field.

To address the limitations of existing deep learning-based fruit and stem segmentation methods, we propose an AS-YOLO model for fruit and stem segmentation. Our work presents four key contributions that distinguish it from previous research.

- We propose a novel AS-YOLO model specifically designed for the segmentation of fruits and stems. This model ensures high processing speed, making it suitable for real-time applications in agricultural automation and fruit-harvesting robots, thereby contributing to enhanced harvest efficiency. The model also incorporates improvements in both accuracy and efficiency over the standard YOLO architecture.
- The AS-YOLO model integrates a ghost bottleneck module to improve computational efficiency by reducing the number of model parameters. This optimization results in faster processing times, achieving real-time performance on an NVIDIA GeForce RTX 3070, with a throughput of 48.78 FPS.
- To overcome the limitations of local feature extraction in conventional YOLO models, we introduce a global attention module (GAM). This module allows the model to better capture the overall context within images, significantly enhancing segmentation accuracy, particularly for smaller objects like stems.
- We constructed and annotated a new dataset, AppleStem-Segmentation (AS-Seg) database, including both public and custom-built data, specifically for the segmentation of apples and their stems. This dataset will be made publicly available to support future research ensuring that future studies can benchmark performance against our work.

The remainder of this paper is organized as follows. Section 2 reviews related works on fruit and stem detection and segmentation. Section 3 describes the proposed AS-YOLO model and its key components. Section 4 presents experimental results, including ablation studies and comparisons with other methods. Finally, Section 5 discusses limitations and future research directions and Section 6 concludes the paper.

## 2. Related Works

Recently, various deep-learning-based methods and classical image processing algorithms have been studied in the detection and segmentation of fruit and stem. Each study improves detection and segmentation performance by addressing environmental factors, such as lighting variations, occlusions, and complex backgrounds, as well as accounting for the diverse shapes and sizes of fruits. Traditional image processing methods utilize color-based models, texture features, and geometric shape information to improve detection performance in complex environments. However, their effectiveness is limited by factors such as lighting variations and fruit shape. Recent deep-learning-based studies have demonstrated robust detection and segmentation performance in various environments using CNNs. However, they have limitations such as high computational cost and difficulty in real-time applications; thus, researchers are investigating lightweight models to address these problems. These studies can be categorized into detailed methods based on each approach.

### 2.1. Fruit Detection Approaches

Various methodologies have been developed for fruit detection, including color-based, texture-based, and multimodal-based methods, with each study improving detection performance by addressing environmental constraints and fruit-specific characteristics. In an early study, RGB-thermal fusion [4] was used because its performance was limited when relying solely on color information. By incorporating thermal channels, this method exhibited more accurate detection and boundary recognition performance under challenging lighting conditions. However, thermal cameras are expensive and are difficult to implement in real-world scenarios. Consequently, ref. [5] we propose a method that enhances edges using Laplacian filters [18] while combining color features, enabling accurate fruit detection in complex backgrounds using only RGB images. This method identifies fruit centers and expands them from the initial regions using a combination of texture and color features. Later studies [6,7] proposed a method that initially detects fruit pixels using only color information without texture features, followed by seed region expansion for more accurate segmentation. However, relying solely on color information is inadequate for capturing essential features, resulting in performance limitations and requiring complex post-processing to achieve accurate results. To address these limitations, studies have proposed extracting and combining various features to improve detection performance. One study introduced the EigenFruit method [8], a principal component analysis (PCA)-based method that applies the EigenFace [19] method for fruit detection. Additionally, ref. [10] proposed a method for detecting fruit regions by extracting texture features using a scale-invariant feature transform (SIFT) [20], speeded-up robust features (SURF) [21], and oriented fast and rotated brief (ORB) [22], subsequently classifying the detected regions using a support vector machine (SVM) [23]. While numerous studies have focused on improving fruit-detection performance across various environments, challenges remain owing to computational costs and variations in fruit size and shape.

### 2.2. Fruit Segmentation Approaches

Fruit segmentation is essential in automated fruit detection systems, enabling precise identification and extraction of fruit shapes. Various studies have been conducted to enhance the segmentation performance, with earlier research proposing a simple method using color histograms [24]. This study proposes a segmentation method that analyzes the color differences between the fruit and background using a color histogram, establishing an optimal threshold for segmentation. However, this study is limited to fruits with distinct colors, such as red, making it difficult to apply in environments where the fruit and background have similar colors. To overcome the limitations, thermal imaging methods [25] using a thermal camera have been proposed to achieve robust performances by segmenting the fruit based on the temperature differences between the object and the background, even when their colors are similar. Furthermore, a hyperspectral imaging [26] method has been proposed to effectively segment fruits and backgrounds by analyzing spectral data using multiple wavelengths. However, thermal and hyperspectral cameras are expensive, which makes their practical applications challenging. Subsequently, deep-learning-based approaches have been introduced to solve these problems. In [27], the study applied Mask R-CNN [14] and improved segmentation performance for elongated fruits by improving anchor boxes and the region proposal network (RPN) [28]. This optimization enables a more accurate detection and segmentation of fruits with elongated shapes. Additionally, attention modules [16] and deformable convolutions [28] are integrated into the Mask R-CNN, demonstrating high accuracy for complex backgrounds. However, the structural limitations of the Mask R-CNN, along with the added modules, make real-time implementation difficult. Consequently, ref. [29] introduced a MobileNetV3 model [30] as the backbone network of the Mask R-CNN, significantly improving the computational efficiency, and the boundary patch refinement (BPR) module was used [31] to improve the segmentation performance. Additionally, the proposed method operates robustly for lighting variations through a multimodule CNN [32] that combines image correction and shape completion modules. However, owing to its complex structure, the computational cost increases, making real-time applications difficult. Given the limitations of previous deep learning methods, which have difficulties operating in real time owing to their complex structures, ref. [33] the YOLOv5-LiNet model was proposed. This model significantly reduces the computational cost by redesigning the network structure of YOLOv5 [34]. Various approaches, from color-based methods to advanced deep learning methods, have been explored to improve the fruit segmentation performance. However, most studies have focused primarily on fruit segmentation, with relatively less emphasis on simultaneous fruit and stem segmentation. Stem segmentation is critical in automated systems, and related studies are discussed in the following section.

### 2.3. Detection and Segmentation Approaches for Fruit and Stem

The detection and segmentation of the fruit and stem is crucial in automated agricultural robots and management systems, with recent studies making significant progress. An earlier study [35] proposed an algorithm to separate fruits from the background by combining the opposition histogram and tiling adaptive (OHTA) color space with an SVM classifier [23]. By using the OHTA color space instead of RGB, this method accentuated the color differences between the object and background, enabling the SVM classifier to detect fruits and stems. However, this method was only effective with simple backgrounds, such as white or black, and its performance decreased significantly with more complex backgrounds. Consequently, this study [36] proposed a method that combines deep-learning-based You Only Look Once version 3 (YOLOv3) [37] with U-Net [38]. After detecting the main objects using YOLOv3, which offered relatively fast detection performance, U-Net was employed to refine the boundary between the fruit and stem for more precise accuracy. This study showed an accuracy of over 80%; however, it required a special lighting system. Subsequently, studies have progressed beyond merely detecting the boundaries or locations of fruits and stems, advancing toward their segmentation of fruits and stems at the pixel level. A study [39] proposed a pixel-level segmentation method using a multi-class SVM to classify pixels as fruits, stems, or backgrounds. It demonstrated high performance on small datasets but struggled to learn complex features, resulting in limited performance on large datasets compared with deep learning-based methods. Later studies, such as [12,13] applied a Mask R-CNN to segment fruits, stems, and backgrounds more effectively. While Mask R-CNN significantly reduced processing time from over 10 s to less than 1 s, making it much faster, it still required excessive time for real-time applications. Other studies have fused RGB and NIR images, utilizing a parallel attention mechanism [40] to fuse the features of both modalities and improve the segmentation performance. A study [11] proposed a method that improves segmentation performance by fusing RGB and NIR images to create multimodal images. Subsequently, it applied a parallel attention mechanism [40] to effectively fuse features between the two modalities. However, this method faced challenges with pixel misalignment between the two image types, leading to reduced accuracy when the images were imperfectly aligned.

Considering the limitations of previous studies, we proposed the AS-YOLO model, which segmented fruits and stems in real-time using only RGB images. Table 1 compares the proposed method with previous studies.

## 3. Materials and Methods

### 3.1. Overview of Proposed Method

**Figure 2:**
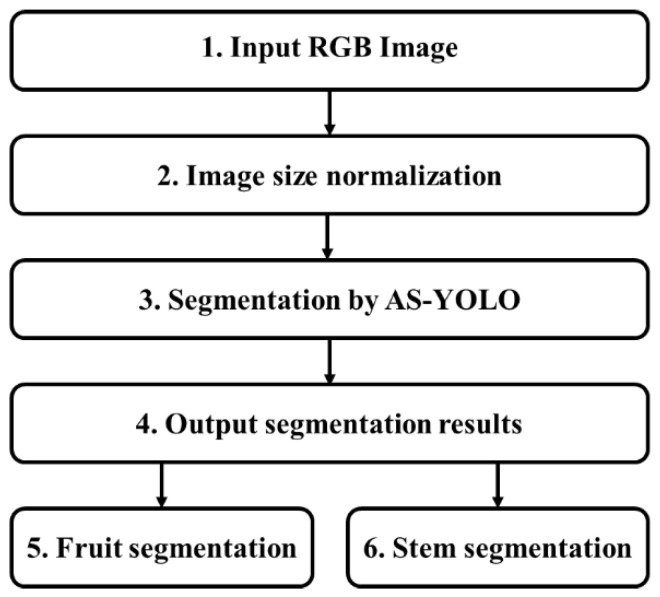
Flowchart of proposed method.

Figure 2 illustrates a flowchart of the proposed method. The input consists of RGB images of apples acquired under various environmental conditions (Step 1 in Figure 2). Given that the input images varied in size, image-size normalization is applied to ensure consistent input dimensions for the model (Step 2 in Figure 2). After image-size normalization, the input image is resized to 640 × 640 (width and height) pixels. The proposed AS-YOLO model segments the fruit and stem (Step 3 in Figure 2). The output of the model consists of segmented results (step 4 in Figure 2), which are presented as binary masks that separate the boundaries and areas of the detected objects (steps 5 and 6 in Figure 2). A detailed description of the AS-YOLO architecture is provided in Section 3.2, while the core components of the model—the ghost bottleneck and global attention module—are explained in Section 3.3 and Section 3.4, respectively.

### 3.2. AS-YOLO Model

**Figure 3:**
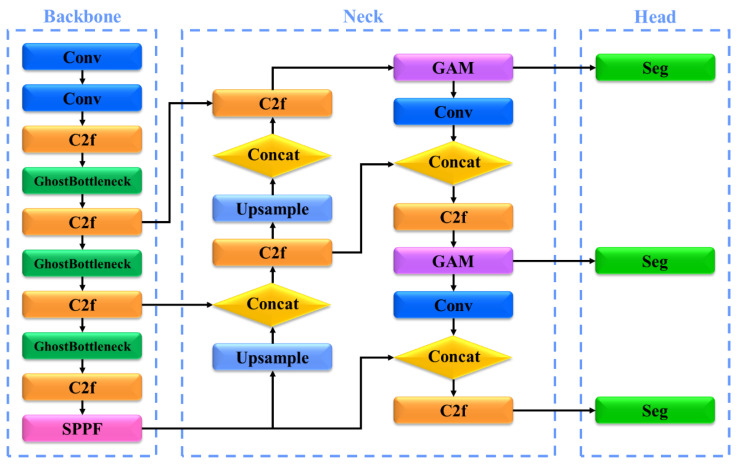
Architecture of AS-YOLO.

The AS-YOLO model is based on the YOLOv8 [42] architecture, with enhanced segmentation performance achieved through the integration of ghost bottlenecks and a GAM. The AS-YOLO model demonstrates both high performance and fast speed for real-time fruit and stem segmentation. The architecture of AS-YOLO is illustrated in Figure 3, with detailed information, such as the kernel, stride, and padding for each layer, provided in Table 2.

The backbone of the AS-YOLO model utilizes the YOLOv8 [42] architecture, incorporating a ghost bottleneck to reduce the computational cost and improve computational efficiency. A ghost bottleneck is a lightweight technique that replaces conventional convolution operations while preserving critical features, reducing the number of parameters, and ensuring real-time performance. The backbone processes input images at multiple resolutions, extracting key features through convolutional layers and ghost bottlenecks to maximize efficiency. A detailed description of ghost bottlenecks is presented in Section 3.3.

The neck of the AS-YOLO model combines a cross-stage partial bottleneck with two convolutions: a faster (C2f) block and a GAM, designed to learn the global features of an image. The GAM enables the model to focus more on key regions of the image, enhancing the segmentation performance of complex objects, such as apples, and smaller objects, such as apple stems. The neck structure combines multi-scale features extracted from the backbone through upsampling and concatenation operations, improving object detection accuracy. A detailed description of the GAM is provided in Section 3.4.

The head of the AS-YOLO model produces the final segmentation results, which include a detailed boundary delineation between the fruit and its stem. The segmentation head is designed to separate the apples and stems with high precision, ensuring distinct class predictions. Additionally, it supports multiclass predictions, enabling the model to accurately detect and segment the boundaries of different objects in a scene. The design of the head ensures that the model can handle fine-grained segmentation of both large and small objects, such as apples and stems.

### 3.3. Ghost Bottleneck

The ghost bottleneck is a critical module in the AS-YOLO model that combines ghost modules with squeeze-and-excitation (SE) layers [43] and depthwise convolution (DWConv) [44] to maximize efficiency. This structure is designed to maintain high performance with low computational cost, making it essential for real-time processing.

**Figure 4:**
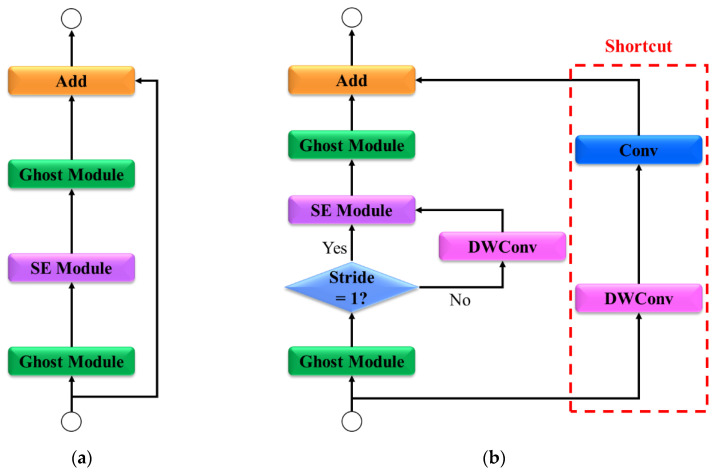
Architecture of ghost bottleneck. (**a**) ghost bottleneck stride = 1 and M ≠ N, (**b**) ghost bottleneck stride = 2 or M ≠ N. SE refers to squeeze-and-excitation, Conv refers to convolution, and DWConv refers to Depth-wise convolution. M indicates the input dimension, while N indicates to the output dimension.

As shown in Figure 4, the ghost bottleneck structure consists of two paths. In the main path, the input is processed by a ghost module that extracts the primary features. In this path, the features are transformed while emphasizing key information. The SE layer is selectively activated based on the stride condition, enhancing the most critical information for each channel. Subsequently, a second ghost module generates additional features, resulting in the final output. In the shortcut path, if the input and output dimensions are identical, an identity connection is used to process the unchanged data. However, if the dimensions differ or the stride is set to two, the data passes through DWConv and a 1 × 1 convolutional layer. The ghost bottleneck combines the outputs from both paths to generate the final output. This design minimizes feature loss while optimizing computational efficiency, establishing it as a critical element for achieving the real-time performance goals of the AS-YOLO network.

The computational process for the ghost bottleneck is mathematically expressed as: In the ghost module, the input feature map X is reduced through a convolutional layer. This step captures the most critical features and produces a smaller feature map $X_{1}$, as shown in Equation (1):

(1) $$X_{1}=\mathrm{Conv}\left(X,c1\to \frac{c2}{r}\right),$$

where c1 and c2 represent the number of input and output channels, respectively, and r represents the filter reduction ratio. Subsequently, additional ghost features are generated through an inexpensive operation typically implemented using depth-wise convolution. Depthwise convolution applies convolution to each individual channel, operating similarly to traditional convolutional layers; however, computational costs are significantly reduced. The newly generated feature map $X_{2}$ is formulated as shown in Equation (2) and combined with $X_{1}$ to form a more expressive feature representation.

(2) $$X_{2}=\mathrm{DWConv}\left(X_{1},\;k,\;s\right),$$

where *k* and *s* represent the kernel size and stride, respectively. The combined output of $X_{1}$ and $X_{2}$ can be expressed as shown in Equation (3). Through this process, expressive features are extracted while maintaining high computational efficiency, without the need for complex convolutional operations.

(3) $$X_{\mathrm{out}}=\left[X_{1},X_{2}\right]$$

If downsampling is required in the ghost bottleneck owing to a stride *s* > 1, DWConv is applied to reduce the spatial resolution while preserving key features. This operation can be mathematically represented as shown in Equation (4), where $X_{\mathrm{out}}$ represents the downsampled feature map.

(4) $$X_{\mathrm{out}}=\mathrm{DWConv}\left(X,\;c1,\;k,\;s\right),$$

where c1 represents the number of channels in the input feature map. This operation adjusts the spatial resolution if necessary. When the stride is one, the operation is skipped, and an identity connection is used instead. The SE module performs squeezing and excitation by adjusting the importance of each channel. During the squeeze phase, global average pooling is applied to each channel to summarize the spatial information. In the excitation phase, the pooled data passes through two fully connected layers where weights are applied, emphasizing the important channels and suppressing the less important ones. The complete process is represented by Equation (5):

(5) $$S=\sigma \left(W_{2}\cdot \delta \left(W_{1}\cdot \mathrm{GAP}\left(X\right)\right)\right),$$

where GAP represents to the global average pooling, $W_{1}\;and\;W_{2}$ represent the weights of the fully connected layer, and δ and σ represent rectified linear unit (ReLU) [45] and sigmoid function, respectively. The importance of each channel was determined using the sigmoid function, and the resulting weights were multiplied by the original feature map to generate the final output $X_{SE}$, as shown in Equation (6):

(6) $$X_{\mathrm{SE}}=X_{\mathrm{out}}\cdot S$$

Subsequently, $X_{\mathrm{SE}}$ was processed by another ghost module to produce the final feature map, as expressed in Equation (7):

(7) $$X_{\mathrm{Ghost}}={GhostModule(X}_{SE})$$

Additional refined features were extracted by reapplying the ghost module. Finally, the output from the ghost module $X_{\mathrm{Ghost}}$, was combined with the output from the shortcut path $X_{\mathrm{Shortcut}}$ to compute the final output, as shown in Equation (8). This residual connection enabled the network to learn effectively without losing important information.

(8) $$X_{final}=X_{Ghost\;}+X_{Shortcut}$$

The final output was generated by combining $X_{Ghost}$, which had passed through the ghost module twice, with an appropriate dimension-matched output from the shortcut path. This process enabled the ghost bottleneck to learn refined and detailed features, contributing to overall network efficiency and enhanced performance.

### 3.4. Global Attention Module

**Figure 5:**
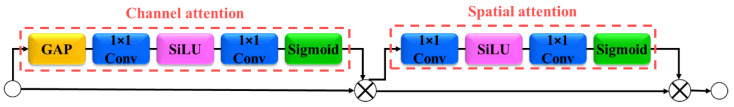
Architecture of global attention module. GAP refers to global average pooling, SiLU refers to sigmoid linear unit, and Conv refers to convolution.

In the AS-YOLO architecture, the GAM is critical in applying attention mechanisms to both the channel and spatial information of the input feature maps, enabling the model to effectively extract important features. As shown in Figure 5, GAM consists of two primary components: channel attention and spatial attention. Each stage analyzes the input data and applies weights to emphasize the critical regions and channels, enabling the model to focus on the most relevant parts of the image and improve the overall segmentation performance.

Additionally, channel attention plays a critical role in assigning importance to each channel in the input feature map. Initially, the input feature map X undergoes spatially compressed in the channel attention mechanism using GAP, which summarizes the spatial information into a single vector. Subsequently, the output passes through two convolutional layers with a sigmoid activation function applied at the end to compute the importance of each channel. Thus, the importance of each channel is determined, and the overall formula for channel attention is expressed in Equation (9):

(9) $$A_{c}=\sigma \left(W_{2}\cdot \delta \left(W_{1}\cdot \mathrm{GAP}\left(X\right)\right)\right),$$

where $W_{1}\;and\;W_{2}$ represent the weights of the convolutional layer, and δ and σ represent sigmoid linear unit (SiLU) [46] activation function and sigmoid function, respectively. The calculated channel attention map $A_{c}$ determines the importance of each channel in the input feature map. Ultimately, feature map $X_{c}$, reflecting channel importance, can be obtained by multiplying channel attention map $A_{c}$ by input feature map X, as shown in Equation (10):

(10) $$X_{c}=X\cdot A_{c}$$

After applying channel attention, GAM applies spatial attention to determine the importance of each spatial location. As shown in Equation (11), the input feature map $X_{c}$ passes through two convolutional layers, followed by the application of a sigmoid function to compute the importance of each spatial position.

(11) $$A_{s}=\sigma \left(W_{4}\cdot \delta \left(W_{3}\cdot X_{\mathrm{chn}}\right)\right),$$

where $W_{3},W_{4}$ represent the weights of the convolutional layer. The spatial attention map $A_{s}$, which represents the importance of each spatial position, is subsequently multiplied by $X_{c}$ to generate the final output $X_{s}$, as expressed in Equation (12):

(12) $$X_{s}=X_{c}\cdot A_{s}$$

By applying attention to both key channels and spatial positions in the input feature map, the GAM enables the model to accurately detect important objects and fine-grained details.

## 4. Experimental Results and Analysis

### 4.1. Experimental Database—AppleStem-Segmentation Database

**Figure 6:**
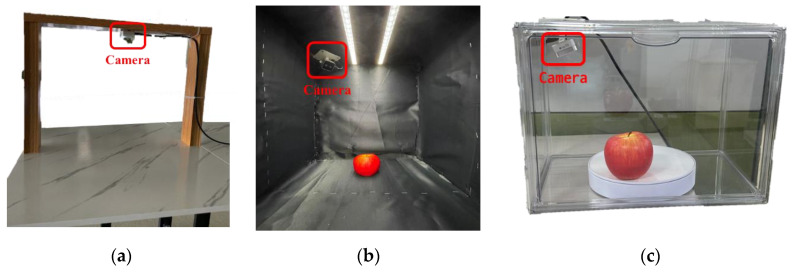
Various experimental setups for apple image acquisition. (**a**) Open table setup, (**b**) Enclosed dark chamber, (**c**) Transparent box with turn table.

In this experiment, we constructed the AS-Seg database by manually labeling portions of the fruit recognition dataset [47,48] along with data from our acquisition process. The camera used for data acquisition was an Intel RealsenseTM D405 [49], which operates optimally at distances ranging from 7 to 50 cm with a field of view (FOV) of 87° × 58° and a maximum resolution of 1280 × 720 pixels. Data acquisition was conducted in three different environments: an open table setup, an enclosed dark chamber setup, and a transparent box with a turntable setup (Figure 6). In the open-table setup, data were collected from various angles of apples placed on a table. In an enclosed dark chamber, the camera captured images in a light-controlled environment to minimize external light interference. Finally, the rotating apples were captured within a transparent acrylic box with a turntable. The images acquired using these settings are shown in Figure 1.

Descriptions of the AS-Seg database used in the experiments are listed in Table 3. The database was divided into training, test, and validation datasets, comprising 70%, 20%, and 10% of the total data, respectively, and used in the model learning, performance evaluation, and final verification stages. Various data-augmentation techniques have been applied to prevent overfitting during the training process, including dropout, mosaic augmentation [50], flipping, and pixel shifting. These augmentations enhanced the robustness of the model by introducing variations into the training data, thereby ensuring better generalization to unseen data.

### 4.2. Training of AS-YOLO

**Figure 7:**
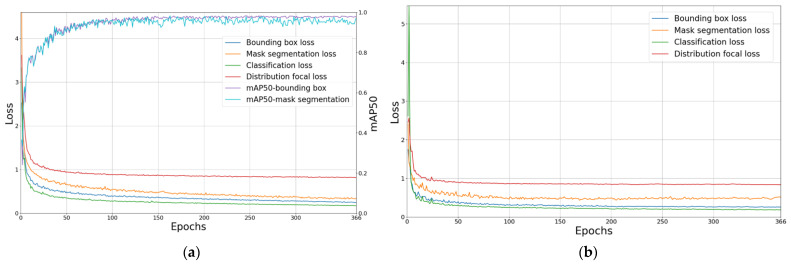
Loss and accuracy curves for training and validation data. (**a**) Loss and accuracy curves for training data, (**b**) Loss curves for validation data.

The AS-YOLO model was trained using training and validation datasets from the AS-Seg dataset. The training was conducted for 500 epochs, and an early stopping technique was applied, which was terminated if the validation loss did not improve for 100 epochs. This method ensured that the model avoided overfitting. The momentum parameter, weight decay, and initial learning rate were set to 0.937, 0.0005, and 0.01, respectively. Figure 7 shows the loss and mAP on the training data, along with the loss on the validation data. The x-axis represents the number of epochs, the left y-axis represents the loss values, and the right y-axis represents the mAP. As the training progressed, the performance of the model in both bounding box and mask segmentation improved steadily. The bounding box, mask segmentation, classification, and distribution focal losses on both the training and validation datasets continuously decreased and converged, indicating effectively learning by the proposed model from the AS-Seg dataset. This confirms the successful training of the AS-YOLO model on the AS-Seg dataset, demonstrating its ability to optimize the performance for both bounding box detection and mask segmentation tasks.

### 4.3. Testing of AS-YOLO

To evaluate the performance of the proposed AS-YOLO model, the key metrics used are precision, recall, mAP@50, and mAP@50–95 for both bounding box and mask segmentation. These performance metrics are crucial in evaluating the accuracy of a model in predicting objects and performing segmentation. Precision refers to the proportion of predicted objects that are correctly detected as true positives. High precision indicates accurate identification of the predicted objects by the model. The recall measures the number of ground-truth objects in a dataset detected by the model. A high recall indicates that the model is effective in detecting most of the objects present in the dataset. mAP@50 calculates the mean average precision across all classes using an intersection over union (IoU) threshold of 0.5. This metric indicates how effectively the model detects objects by measuring the overlap between the predicted and ground truth objects, typically using the IoU as a threshold for accuracy. mAP@50–95 averages precision across multiple IoU thresholds from 0.5 to 0.95 in steps of 0.05. This serves as a more stringent evaluation, testing the consistency and robustness of the model for detecting objects under varying conditions. These metrics are used to compare the performance of the AS-YOLO model with those of other methods in both object detection and segmentation tasks.

### 4.4. Ablation Studies

To evaluate the contributions of the proposed modules to the performance of YOLOv8, we conducted an ablation study. The models were trained and evaluated on the apple and stem segmentation under consistent experimental conditions, with performance measured using precision, recall, mAP@50, and mAP@50–95. The results of the ablation study are presented in Table 4, which provides performance comparisons of different module configurations.

The baseline YOLOv8 model achieved an overall mAP@50 of 0.935 and mAP@50–95 of 0.757. While the model performed well on apple segmentation, its performance on stem segmentation was comparatively lower, likely due to challenges such as small object size and occlusions.

Incorporating Ghost bottleneck into YOLOv8 improved the overall mAP@50 to 0.941 and mAP@50–95 to 0.761. Notably, precision of stem segmentation increased significantly from 0.886 to 0.939, demonstrating the ability of the Ghost bottleneck to enhance computational efficiency while improving segmentation accuracy for complex features.

Adding the GAM module to YOLOv8 significantly improved recall and mAP@50–95, with this improvement attributed to GAM module’s ability to capture global contextual information, particularly benefiting stem segmentation.

The combination of ghost bottleneck and GAM in AS-YOLO achieved the best overall performance, with an mAP@50 of 0.956 and mAP@50–95 of 0.782. The model maintained high precision and recall for apple segmentation while significantly improving the balance between precision and recall for stem segmentation.

### 4.5. Comparisons with Other Methods

This study compared the performance of the proposed model with state-of-the-art methods, such as Mask R-CNN [14], YOLACT [51], HTC [52], YOLOv5 [34], PointRend [53], and YOLOv8 [42], YOLOv11 [54]. Table 5 presents a comparison of the detection and segmentation performances, and the inference speeds of the test set. The results demonstrated that the proposed model outperformed the other methods across most metrics while achieving the fastest inference speed. We primarily conducted experiments using YOLO-based methods, as they have consistently demonstrated high performance across various domains [55].

The state-of-the-art methods performed reasonably well for the apple class, achieving high precision and recall across various architectures. However, there was a clear performance gap between these models and the AS-YOLO models, particularly for the stem class. AS-YOLO achieved the highest mAP@50–95 score of 0.674 for bounding box detection in the stem class, outperforming the scores of YOLOv8 at 0.597 and YOLOv11 at 0.664. This highlight its superior ability to segment small objects like stems, whereas other models encountered challenges.

AS-YOLO also demonstrated the highest speed among the compared methods, processing at 129.8 frames per second. This was approximately 13 percent faster than YOLOv8, which processed at 114.9 frames per second, and 16 percent faster than YOLOv11, which achieved 111.4 frames per second. Methods like Mask R-CNN and PointRend, with speeds of 49.5 and 57.2 frames per second respectively, lagged significantly due to their heavier architecture and reliance on iterative processes that are less suited for real-time tasks.

For the apple class, AS-YOLO achieved outstanding precision and recall, reaching values of 0.999 and 1.0 respectively. Its performance for the stem class was similarly impressive, with a precision of 0.888 and an mAP@50–95 of 0.574. In comparison, YOLOv11 showed competitive results, achieving an overall mAP@50–95 of 0.827 for bounding box detection, but it still lagged behind AS-YOLO in segmenting small objects such as stems. One of the reasons for YOLOv11’s lower performance on small object segmentation can be attributed to its increased computational complexity due to the integration of advanced transformer modules and adaptive spatial fusion mechanisms, as discussed in [56]. While these additions improve contextual understanding and feature fusion, they can introduce challenges in detecting fine-grained structures such as stems due to increased inference time and higher model capacity requirements. Moreover, YOLOv11 exhibited a slight decline in recall, which may indicate that it struggles to generalize well on smaller object classes with limited representation.

The effective balance between accuracy and speed in AS-YOLO is primarily achieved through the incorporation of the GAM module and ghost bottleneck structures. These components enhance global feature extraction and minimize computational overhead, enabling AS-YOLO to process both large and small objects efficiently. This makes it a practical solution for real-time applications, outperforming more complex methods such as Mask R-CNN.

In conclusion, AS-YOLO outperforms other state-of-the-art methods by providing exceptional accuracy for small object detection while maintaining real-time performance.

**Figure 8:**
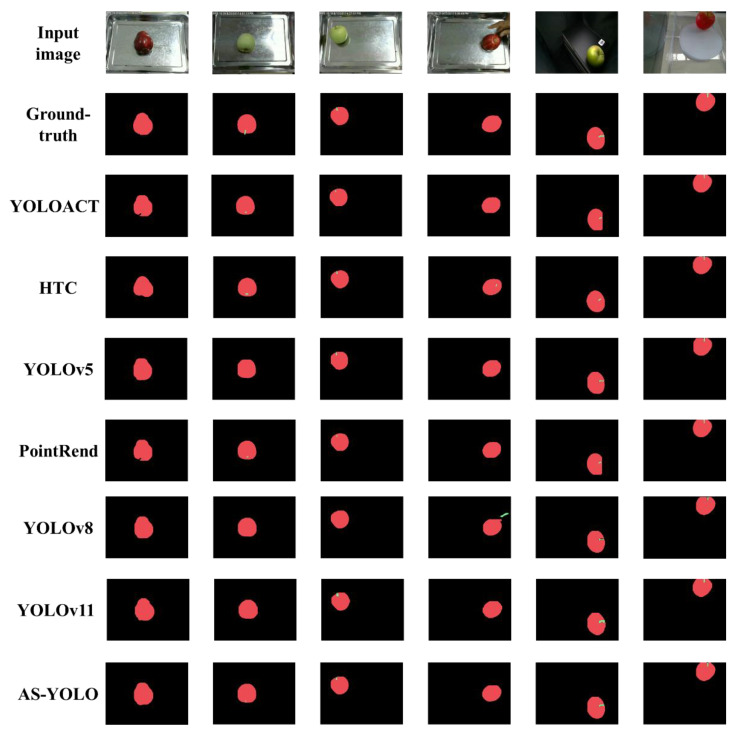
Comparison of segmentation result images in AS-Seg dataset.

Figure 8 presents the segmentation results compared with other state-of-the-art method. The result shows that the proposed AS-YOLO model in this paper effectively segments the stem area overall, with no false positives of the hand being misidentified as a stem. The results demonstrate that the proposed method achieves robust performance in the fruit class, with particularly strong results in the segmentation of the stem class. This demonstrates that the AS-YOLO model achieves superior numerical performance, with its visualized results indicating the effectiveness of the model.

## 5. Limitations and Future Works

In our study, we proposed the AS-YOLO model to detect and segment apple and stem regions in 2D images. Although the performance of the proposed AS-YOLO model was confirmed to be higher than that of the existing method, there are some limitations in applying it to the actual apple post-harvest processing system.

First, while the AS-YOLO model demonstrates strong performance on the AppleStem-Segmentation database, its generalization to other fruit types or datasets with significantly different environmental conditions has not been extensively evaluated. Second, when applied to an actual apple post-processing automation system, there is a problem that it is difficult to precisely recognize and remove the stem based only on the detection results on the 2D image.

Considering these points, future research will focus on enhancing the generalization capabilities of the AS-YOLO model to ensure robust performance across diverse fruit types and varying environmental conditions. This will involve expanding the dataset to include a wider range of fruit species and conditions. In addition, based on the results of this study, we plan to study a deep learning model that estimates the actual 3D coordinates of the apple and stem using 3D point cloud data to apply to the apple post-processing automation system.

## 6. Conclusions

We propose the AS-YOLO model as an efficient model for fruit and stem segmentation, facilitating the stem removal process by fruit-harvesting robots. By integrating ghost bottleneck and global attention modules into the YOLOv8 architecture, the proposed method achieves improved computational efficiency and enhanced detection accuracy, particularly for small objects such as stems. The experimental results demonstrate that the proposed AS-YOLO model achieves real-time inference performance at 129.8 FPS on a GPU while maintaining high accuracy, particularly in metrics such as mAP @50 and mAP @50–95. Compared with previous models, AS-YOLO provides faster and more accurate performance, highlighting its potential for real-time automation systems.

This research contributes to the automation of stem removal in fruit harvesting and provides a foundational technology that can be applied to other agricultural automation tasks. Future work will focus on extending the model to 3D image-based segmentation, enabling a more precise representation of the fruit shape, size, and depth. Additionally, we will enhance the robustness of the model in agricultural environments with varying lighting conditions, complex backgrounds, and obstacles, ensuring consistent performance under these challenging scenarios.

## Figures and Tables

**Table 1:** Comparison of previous researches and proposed method.

Category		Method	Advantage	Limitation
Fruit	Detection	RGB-thermal fusion [4]	Combining color and temperature information to provide robust detection performance even in challenging lighting conditions	The high cost and installation challenges of thermal cameras limit their practical use in real agricultural environments
		Color-based [6,7]	Detection based on color features, offering low computational cost and suitability for real-time detection	Performance degradation in cases of similar-colored backgrounds
		Texture + color-based [5]	Accurate separation of fruit boundaries in complex backgrounds through the combination of texture and color information	High computational cost and difficulty in real-time implementation
		EigenFruit + gabor filter [8]	Effective extraction of complex texture features through the application of the PCA-based EigenFruit technique and Gabor filtering	High computational cost and the need for high-resolution images make PCA-based methods unsuitable for real-time applications
		Color feature extraction [9]	Robust performance with minimal post-processing through efficient use of color features.	Decreased detection accuracy in complex backgrounds or occluded fruits
		Texture-based [10]	Robust detection performance in backgrounds with similar colors by using texture features such as SIFT, SURF, and ORB	High computational costs and insufficient texture features in small fruits, leading to performance limitations
	Segmentation	Color histogram [24]	Effective separation of fruit and background under various lighting conditions using color histograms	Performance degradation in cases of color-similar backgrounds or complex environments.
		Thermal imaging [25]	High performance in complex lighting conditions or color-similar backgrounds by using thermal imaging	Difficulty in real-world application due to the low resolution and high installation cost of thermal camera
		Hyperspectral imaging + LDA [26]	Effective separation of fruit and background in challenging scenarios where only RGB struggles, using hundreds of wavelength bands	High hyperspectral equipment, computational, and data processing costs
		Mask R-CNN [27]	High performance across complex backgrounds and various object sizes through ROIAlign and multi-stage architecture	High computational cost and difficulty in real-time application due to the two-stage structure
		Multi-module CNN [32]	Robust segmentation performance in varying lighting conditions and occlusions through the integration of multiple modules	High computational cost due to the complex model structure, limiting real-time application
		Mask R-CNN + attention module [41]	High accuracy in complex backgrounds and occlusion through the integration of attention mechanism and deformable convolution	Increased computational cost and difficulty in real-time implementation due to the complexity of the module
		Mask R-CNN + BPR [29]	Accurate pixel-level segmentation in complex environments enabled by the BPR module	Real-time performance limitations due to the two-stage structure and anchor dependency affecting fruit shapes
		YOLOv5-LiNet [33]	Capacity for real-time implementation due to the lightweight structure	Performance limitations in handling complex fruit shapes or intricate backgrounds
Fruit + Stem	Detection	OHTA + SVM [35]	Enhanced color differentiation using the OHTA color space, effectively distinguishing the boundaries between the fruit and stem	Performance limitations in complex backgrounds, with robustness confined to specific color ranges
		YOLOv3 + U-Net [36]	Maintenance of high accuracy under various lighting conditions through the combination of YOLOv3 and U-Net	Increased costs due to the need for specialized lighting equipment
	Segmentation	Multi-class SVM [39]	High performance on small-scale datasets	Limited performance in scenarios requiring learning of complex features and in cases with large datasets
		Mask R-CNN [12,13]	High performance achieved across complex backgrounds and varying object sizes through ROIAlign and a multi-stage architecture.	Increased computational load, resulting in reduced image processing speed and limitations in real-time application
		YOLACTFusion [11]	Fusion of RGB and NIR images enabling high performance even in backgrounds with similar colors	Performance degradation and significant alignment errors when the two images are not properly aligned
		AS-YOLO (Proposed method)	Operation using only an RGB camera without additional specialized equipment, enabling real-time processing and enhancing segmentation performance through the integration of the global attention mechanism	Reduced segmentation accuracy for stems compared with fruits

**Table 2:** Structure of Figure 3. Conv refers to convolutional layer, C2f refers to cross stage partial bottleneck with 2 convolutions—faster, SPPF refers to spatial pyramid pooling fusion, and GAM refers to global attention mechanism.

Layer Type		Number of Filters	Size of Kernel	Number of Strides	Number of Padding	Number of Iteration	Input
Input layer		-	-	-	-	-	Image
Back bone	Conv1	64	3 × 3	2	1	1	Input layer
	Conv2	128	3 × 3	2	1	1	Conv1
	C2f block1	128	3 × 3	-	-	3	Conv2
	GhostBottleneck1	256	3 × 3	2	1	1	C2f block1
	C2f block2	256	3 × 3	-	-	6	GhostBottleneck1
	GhostBottleneck2	512	3 × 3	2	1	1	C2f block2
	C2f block3	512	3 × 3	-	-	6	GhostBottleneck2
	GhostBottleneck3	1024	3 × 3	2	1	1	C2f block3
	C2f block4	1024	3 × 3	-	-	3	GhostBottleneck3
	SPPF block	1024	5 × 5	-	1	1	C2f block4
Neck	Upsample1	-	-	2	-	1	SPPF block
	Concat1	-	-	-	-	1	Upsample1, C2fblock3
	C2f block5	512	3 × 3	-	-	3	Concat1
	Upsample2	-	-	2	-	1	C2f block5
	Concat2	-	-	-	-	1	Upsample2, C2fblock2
	C2f block6	256	3 × 3	-	-	3	Concat2
	GAM block1	-	-	-	-	1	C2f block6
	Conv3	256	3 × 3	2	1	1	GAM block1
	Concat3	-	-	-	-	1	Conv3, C2f block5
	C2f block7	512	3 × 3	-	-	3	Cpmcat3
	GAM block2	-	-	-	-	1	C2f block7
	Conv4	512	3 × 3	-	-	1	GAM block2
	Concat4	-	-	-	-	1	Conv4, SPPF block
	C2f block8	1024	3 × 3	-	-	1	Concat4
Segmentation head		-	-	-	-	-	GAM block1, GAM block2, C2f block8

**Table 3:** Description of AS-Seg database.

AS-Seg Database	Train	Validation	Test
Open table setup	196	28	56
Enclosed dark chamber	83	11	23
Transparent box with turn table	182	26	52
Open database	421	61	121
Overall	882	126	252

**Table 4:** Performance comparisons of ablation studies.

Methods	Class	Precision	Recall	mAP@50	mAP@50–95
YOLOv8	All	0.942	0.886	0.935	0.757
	Apple	0.998	**1**	**0.995**	**0.99**
	Stem	0.886	0.771	0.875	0.523
YOLOv8 + Ghost	All	**0.969**	0.869	0.941	0.761
	Apple	**0.999**	**1**	0.995	0.985
	Stem	**0.939**	0.737	0.888	0.536
YOLOv8 + GAM	All	0.9	**0.948**	0.954	0.772
	Apple	0.998	**1**	**0.995**	0.989
	Stem	0.803	**0.897**	0.913	0.555
YOLOv8 + GAM + Ghost (AS-YOLO)	All	0.943	0.932	**0.956**	**0.782**
	Apple	**0.999**	**1**	**0.995**	0.989
	Stem	0.888	0.864	**0.916**	**0.574**

**Table 5:** Performance comparisons of detection and segmentation methods on the test set.

Methods	Class	Bounding Box				Mask				FPS
		Precision	Recall	mAP @50	mAP @50–95	Precision	Recall	mAP@50	mAP @50–95	
Mask R-CNN [14]	All	0.82	0.959	0.942	0.762	0.82	0.959	0.928	0.743	49.5
	Apple	**1**	**1**	0.99	0.97	**1**	**1**	0.99	0.985	
	Stem	0.64	0.917	0.893	0.553	0.64	0.917	0.866	0.5	
YOLACT [51]	All	0.876	0.897	0.913	0.536	0.876	0.897	0.83	0.584	51.9
	Apple	0.99	**1**	0.988	0.731	0.99	**1**	0.988	0.937	
	Stem	0.762	0.793	0.827	0.341	0.762	0.793	0.671	0.231	
HTC [52]	All	0.828	0.945	0.938	0.778	0.828	0.945	0.932	0.737	52.9
	Apple	**1**	**1**	0.989	0.981	**1**	**1**	0.989	0.987	
	Stem	0.656	0.89	0.886	0.575	0.656	0.89	0.874	0.486	
YOLOv5 [34]	All	0.748	0.763	0.8	0.482	0.763	0.775	0.813	0.586	71.4
	Apple	0.859	**1**	**0.995**	0.665	0.859	**1**	**0.995**	0.868	
	Stem	0.637	0.525	0.606	0.3	0.667	0.551	0.631	0.304	
PointRend [53]	All	0.836	**0.98**	**0.96**	0.765	0.836	**0.98**	0.936	0.762	57.2
	Apple	**1**	**1**	0.99	0.957	**1**	**1**	0.99	0.987	
	Stem	0.671	**0.959**	**0.928**	0.573	0.671	**0.959**	0.881	0.536	
YOLOv8 [42]	All	0.94	0.89	0.949	0.795	0.942	0.886	0.935	0.757	114.9
	Apple	0.998	**1**	**0.995**	**0.992**	0.998	**1**	**0.995**	0.99	
	Stem	0.881	0.78	0.903	0.597	0.886	0.771	0.875	0.523	
YOLOv11 [54]	All	0.921	0.939	0.958	0.827	0.913	0.93	0.942	0.752	111.4
	Apple	0.997	**1**	**0.995**	0.99	0.997	**1**	**0.995**	**0.991**	
	Stem	0.845	0.878	0.921	0.664	0.829	0.86	0.889	0.512	
Proposed method (AS-YOLO)	All	**0.943**	0.932	**0.96**	**0.833**	**0.943**	0.932	**0.956**	**0.782**	**129.8**
	Apple	0.999	**1**	**0.995**	0.991	0.999	**1**	**0.995**	0.989	
	Stem	**0.888**	0.864	0.924	0.674	**0.888**	0.864	**0.916**	**0.574**	

## Data Availability

The datasets generated and/or analyzed during the current study will be made publicly available on GitHub upon acceptance of this manuscript for publication. The repository can be accessed at: https://github.com/BAN2ARU/AS-YOLO (accessed on 18 February 2025). To comply with data sharing requirements and transparency, a data usage agreement will be provided on the GitHub repository, and all users are requested to acknowledge and adhere to this agreement when accessing the data.
